# Femtosecond time-resolved X-ray absorption spectroscopy of anatase TiO_2_ nanoparticles using XFEL

**DOI:** 10.1063/1.4989862

**Published:** 2017-06-30

**Authors:** Yuki Obara, Hironori Ito, Terumasa Ito, Naoya Kurahashi, Stephan Thürmer, Hiroki Tanaka, Tetsuo Katayama, Tadashi Togashi, Shigeki Owada, Yo-ichi Yamamoto, Shutaro Karashima, Junichi Nishitani, Makina Yabashi, Toshinori Suzuki, Kazuhiko Misawa

**Affiliations:** 1 Department of Applied Physics, Tokyo University of Agriculture and Technology, 2-24-16 Naka-cho, Koganei, Tokyo 184-8588, Japan; 2 Institute of Global Innovation Research, Tokyo University of Agriculture and Technology, 2-24-16 Naka-cho, Koganei, Tokyo 184-8588, Japan; 3 Interdisciplinary Research Unit in Photon-Nano Science, Tokyo University of Agriculture and Technology, 2-24-16 Naka-cho, Koganei, Tokyo 184-8588, Japan; 4 Department of Chemistry, Graduate School of Science, Kyoto University, Kitashirakawa-Oiwakecho, Sakyo-ku, Kyoto 606-8502, Japan; 5 Japan Synchrotron Radiation Research Institute, 1-1-1 Kouto, Sayo-cho, Sayo-gun, Hyogo 679-5198, Japan; 6 RIKEN SPring-8 Center, 1-1-1 Kouto, Sayo-cho, Sayo-gun, Hyogo 679-5148, Japan

## Abstract

The charge-carrier dynamics of anatase TiO_2_ nanoparticles in an aqueous solution were studied by femtosecond time-resolved X-ray absorption spectroscopy using an X-ray free electron laser in combination with a synchronized ultraviolet femtosecond laser (268 nm). Using an arrival time monitor for the X-ray pulses, we obtained a temporal resolution of 170 fs. The transient X-ray absorption spectra revealed an ultrafast Ti K-edge shift and a subsequent growth of a pre-edge structure. The edge shift occurred in ca. 100 fs and is ascribed to reduction of Ti by localization of generated conduction band electrons into shallow traps of self-trapped polarons or deep traps at penta-coordinate Ti sites. Growth of the pre-edge feature and reduction of the above-edge peak intensity occur with similar time constants of 300–400 fs, which we assign to the structural distortion dynamics near the surface.

## I. INTRODUCTION

Ahmed H. Zewail and his colleagues' demonstration of a “real-time” visualization of nuclear motion in molecular systems via ultrafast spectroscopy in 1987 opened up a new era of Femtochemistry. The Nobel prize for Chemistry was awarded to Zewail in 1999 for this achievement.^1,2^ However, ultrafast laser spectroscopy in the ultraviolet (UV), visible, and infrared regions does not necessarily provide the full information about atomic-scale structural dynamics. Thus, Zewail has further extended his work using time-resolved electron diffraction. An alternative approach to access structural dynamics is ultrafast X-ray absorption/diffraction spectroscopy. In this contribution, we describe time-resolved X-ray absorption spectroscopy (TR-XAS)^3–10^ of Titanium dioxide (TiO_2_) nanoparticles in an aqueous solution.

Titanium dioxide has widespread applications in many chemical and industrial processes such as deodorization, antifouling, sterilization, disinfection, and hydrogen generation from water.^11,12^ TiO_2_ nanoparticles exhibit high photocatalytic activity for water splitting and decomposition of environmental pollutants and bacteria. The photocatalytic activity of TiO_2_ under UV radiation stems from the promotion of an electron from the valence band to the conduction band and consequent transport of the electron and hole to the TiO_2_ surface. However, the understanding of the underlying mechanistic details of charge transport and trapping is still lacking. It is necessary to elucidate the transport and trapping dynamics involved in the photocatalytic activity of TiO_2_.

So far, transport and trapping dynamics in TiO_2_ nano-particles have been studied via transient absorption spectroscopy (TAS) in the visible and near-infrared region. Photoexcitation of TiO_2_ has a broad transient absorption spectrum extending from visible to near infrared, in which the signals of trapped electrons and trapped holes have been identified.^13–19^ Yang and Tamai have studied anatase TiO_2_ nanoparticles in an aqueous solution using TAS, and they found that 360 nm photoexcitation creates an immediate rise of the photoabsorption signal at 520 nm; the estimated time constant was shorter than 50 fs.^15^ Since this signal disappeared by addition of SCN^−^, a well-known scavenger of holes, into the solution, Yang and Tamai concluded that the absorption at 520 nm must be due to trapped holes at the surface and that these holes were transferred to SCN^−^ on an ultrashort timescale. On the other hand, absorption at 700 nm was assigned to trapped electrons at Ti^3+^ sites, and the trapping time scale was estimated to be 260 fs.^15^ In similar experiments on nanocrystalline films of TiO_2_, Furube and colleagues have found that 355 nm excitation created a trapped electron in less than 100 fs, whereas 266 nm excitation increased the electron trapping time to 150–250 fs.^18^ These immediate electron traps were assigned as shallow traps near the surface, whereas relaxation to deep (bulk) traps was found to occur in hundreds of picoseconds. The difference in the transport and trapping times of electrons and holes was ascribed to the difference in their effective masses: 0.8 *m*_e_ of a hole and 10 *m*_e_ of an electron, where *m*_e_ is the mass of a free electron.^20^ It has been argued that the electrons in the conduction band are trapped within a traveling distance of several nanometers after their creation, while holes are transferred to the interface very rapidly. The latter plays an important role in photocatalytic oxidation activity of TiO_2_.^11,12^

TR-XAS is well suited for the study of charge-carrier dynamics, because it enables real-time and direct observation of the oxidation state of Ti. For anatase TiO_2_ nanoparticles, Chergui and colleagues have performed TR-XAS using synchrotron radiation.^9,10^ They reported two types of experiments, with and without the laser-electron slicing technique. Without the slicing, a synchrotron radiation facility produces X-ray pulses with a duration of tens of picoseconds. The laser-electron slicing technique introduces a femtosecond laser in the synchrotrons storage ring to create a thin slice of an electron bunch that emits a femtosecond X-ray pulse.^21,22^ This, however, reduces the X-ray photon flux significantly, which makes TR-XAS using this laser-electron slicing technique highly challenging. Chergui and colleagues observed that UV photoexcitation of anatase TiO_2_ induces a red-shift of the Ti K edge and a significant enhancement of the pre-edge region, both of which occurred within their time-resolution and persisted over sub-nanoseconds.^9^ The red-shift of the K-edge is indicative of a reduction of the titanium (Ti^4+^ → Ti^3+^), and the enhanced pre-edge peak has been ascribed to electron trapping at penta-coordinated Ti atoms.^23,24^ In the follow-up femtosecond study, the time scale of the edge shift was measured more precisely to be less than 300 fs, although the signal to noise ratio was severely limited by an extremely low photon flux due to the laser-electron slicing employed.^10^ The low photon flux has been prohibitive to similar laser-electron slicing measurements for the pre-edge peak.

X-ray free electron lasers (XFELs) create intense short pulses in the X-ray region, which opened up new avenues in ultrafast spectroscopy of electronic and structural dynamics in solutions and interfaces. In this work, we present femtosecond TR-XAS of TiO_2_ nanoparticles suspended in an aqueous solution measured with the SPring-8 Angstrom Compact Free Electron Laser (SACLA)^25^ in combination with a synchronized femtosecond laser.^26^ We employ an arrival-time monitor of X-ray pulses to make full use of the SACLA's ultrashort pulses,^27,28^ and we determine the time constants for the K-edge shift and the growth of the pre-edge peak accurately.

## II. EXPERIMENT

### A. Sample

**FIG. 1.**
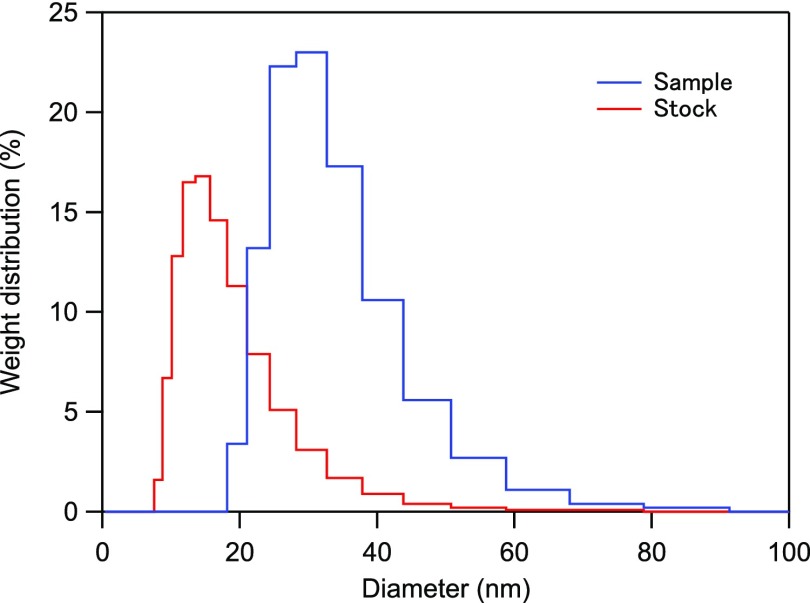
Weight distributions of the particle diameter in the stock solution (red line) and the sample (blue line; stock solution diluted to 50 mM) were measured by dynamic light scattering. The measured particle size ranges were 10 to 20 nm and 20 to 40 nm, respectively.

We prepared an aqueous 50 mM solution of TiO_2_ nanoparticles by diluting a concentrated stock solution (TAYCA, TKS-201) of 33 wt. % with pure water. The crystal structure and the mean diameter of the primary particles were determined by X-ray diffraction (Fig. Suppl-1 in the supplementary material) to be anatase and about 7 nm, respectively. The pH value of the sample solution was 2.0. Note that the surface of the TiO_2_ nanoparticles in the aqueous solution is electrically neutral at pH = 5–7,^29^ while being charged positively at lower pH and negatively at higher pH.^30^ The size distributions of the TiO_2_ particles in the stock solution and the prepared sample were measured by dynamic light scattering (DLS) using a Malvern Zetasizer Nano ZS. The results are shown in Fig. 1. The measured particle size ranges were 10–20 nm and 20–40 nm, respectively. These particles, often denoted as agglomerates, are composed of loosely bound primary particles.^31,32^

For the XFEL experiment, the sample solution was ejected from a fused silica capillary forming a cylindrical liquid jet of 100 *μ*m in diameter. A flow rate of 2.82 ml/min was maintained by a tube pump (Master Flex I/P 77601-10). The used solution was discarded and not recirculated.

### B. Total X-ray fluorescence yield method

SACLA delivers high-intensity X-ray pulses with an estimated time duration of less than 10 fs at a repetition rate of 30 Hz.^25,26^ The intrinsically broad X-ray photon energy distribution was centered near the Ti K-edge by adjusting the conditions of the accelerator and undulator of SACLA, and a Si (111) X-ray monochromator was employed to monochromatize the energy distribution down to a bandwidth of 1.3 eV (FWHM) and to scan the photon energy. The monochromatized probe X-ray was focused on the sample solution by beryllium compound refraction lenses. The resulting focal beam diameter was 20 *μ*m. A small fraction of the X-ray pulse was sampled using a Kapton film to monitor its intensity. The X-ray absorption spectra were measured by monitoring the total intensity of X-ray fluorescence^5,8^ with a photodiode (an active area of 10×10 mm^2^; placed 7 mm away from the sample) while scanning the monochromator. The sample and the photodiode were placed in a box filled with Helium gas in order to prevent X-ray light attenuation in air.

The X-ray fluorescence intensity *I*_F_ is expressed as a function of the X-ray photon intensity *I*_0_ as follows:

$$I_{F}\left(E\right)=\;C\cdot \sigma_{\text{abs}}\left(E\right)\Phi_{\text{fluor}}\Phi_{\text{det}}I_{0}\left(E\right),$$

where *E*, *C*, $\sigma_{\text{abs}}(E)$, $\Phi_{\text{fluor}}$, $\Phi_{\text{det}}$, and *I*_0_(*E*) are the photon energy, a constant factor, the absorption cross-section, the fluorescence quantum yield, the detection quantum yield, and the X-ray pulse intensity, respectively. In practice, the intensity and spectral shape of the X-ray pulses fluctuate on a shot-to-shot basis because of the nature of the self-amplified stimulated emission (SASE) process of SACLA. We recorded the fluorescence intensity *I*_F_(*E*) for every shot together with X-ray pulse intensity *I*_0_(*E*) and normalized the former with the latter on a shot-to-shot basis

$$\frac{I_{F}}{I_{0}}=\;C\cdot \sigma_{\text{abs}}(E)\Phi_{\text{fluo}r}\Phi_{\text{det}}\;.$$

The detectors are confirmed to have a good linearity performance. We determined the absorption cross-section by averaging the normalized fluorescence intensity over a sufficient number of shots.

### C. Time-resolved X-ray absorption spectroscopy

We excited the sample with 268-nm UV light which was the third harmonic (TH) of a synchronized Ti:Sapphire amplified laser. The TH generation was performed in two steps, second harmonic (SH) generation of the fundamental Ti:Sapphire output and sum-frequency generation between the fundamental and second harmonic. We used two β-BaB_2_O_4_ (BBO) crystals: one was a type I crystal (θ = 29.2°, ϕ = 90°) with a thickness of 0.5 mm for the second harmonic generation, while the other was a type I crystal (θ = 44.3°, ϕ = 90°) with a thickness of 0.5 mm for the sum-frequency generation. The 0.5-mm thick BBO crystals enabled us to generate powerful UV light with a maximum energy of 0.3 mJ. The pulse width of UV light was estimated to be ∼170 fs by cross-correlation measurement with the fundamental. The rather long duration originates from the temporal broadening of SH due to the group velocity mismatch between the fundamental and SH in the first BBO crystal.

**FIG. 2.**
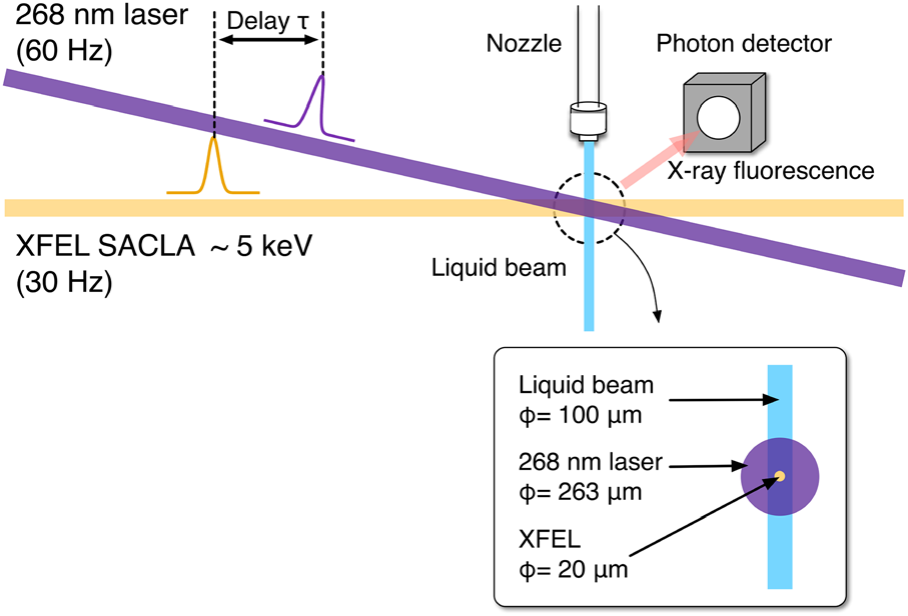
Sketch of the time-resolved X-ray absorption detection scheme used in this study. The crossing angle between the k-vector of the 268 nm laser and the XFEL was less than 10°. The inset shows the schematic of the spatial overlap between the 268 nm laser, XFEL, and sample liquid beam.

Figure 2 schematically shows our experimental setup for TR-XAS. The 60 Hz pulse train of the UV light was reduced to 30 Hz using a chopper wheel and focused on the liquid jet using a lens with a focal length of 400 mm. Since a long light path from the Ti:Sapphire laser to the sample made the pointing of UV light unstable, we set the UV beam diameter to be greater than 100 *μ*m to stabilize sample illumination. The spatial profile of the UV light at the sample position was characterized to be a Gaussian distribution with an effective spot diameter of 263 *μ*m using a beam profiler. The transient X-ray absorption intensity varied linearly with the UV pulse energy up to 140 *μ*J [compare the energy dependence in Fig. 7(a)]. Consequently, spectroscopic scans were performed much below this limit at a UV pulse energy of 95 *μ*J.

The timing of the 268 nm pulses with respect to the X-ray pulses was adjusted using an electronic delay circuit and subsequently fine-tuned using a computer-controlled linear translation stage in the UV beam path. During data acquisition, the delay time τ was scanned between –2 ps and 8 ps in a stop-and-go manner while recording 1000 shots at every step.

### D. Arrival time diagnostics

We have initially performed TR-XAS of TiO_2_ nanoparticles without the arrival timing diagnostics, but we revisited the experiment including the diagnostics to ascertain the observed time constants. The latter provided superior time-resolution, so that we mainly present the results obtained including the arrival time diagnostics in this paper. The pulse energy diverted for the timing measurements is only less than 3% of the total X-ray pulse energy of SACLA. Although XFEL sources provide femtosecond X-ray pulses, typically ca. 10 fs in width, the achieved time-resolution in pump-probe experiments is degraded by short-term jitters and long-term drifts of the arrival timings between X-ray and pump-laser pulses. To improve the time resolution in pump-probe experiments up to the limit determined by the single-shot cross-correlation time, a post-process analysis combined with the arrival-time diagnostics explained below was employed.

**FIG. 3.**
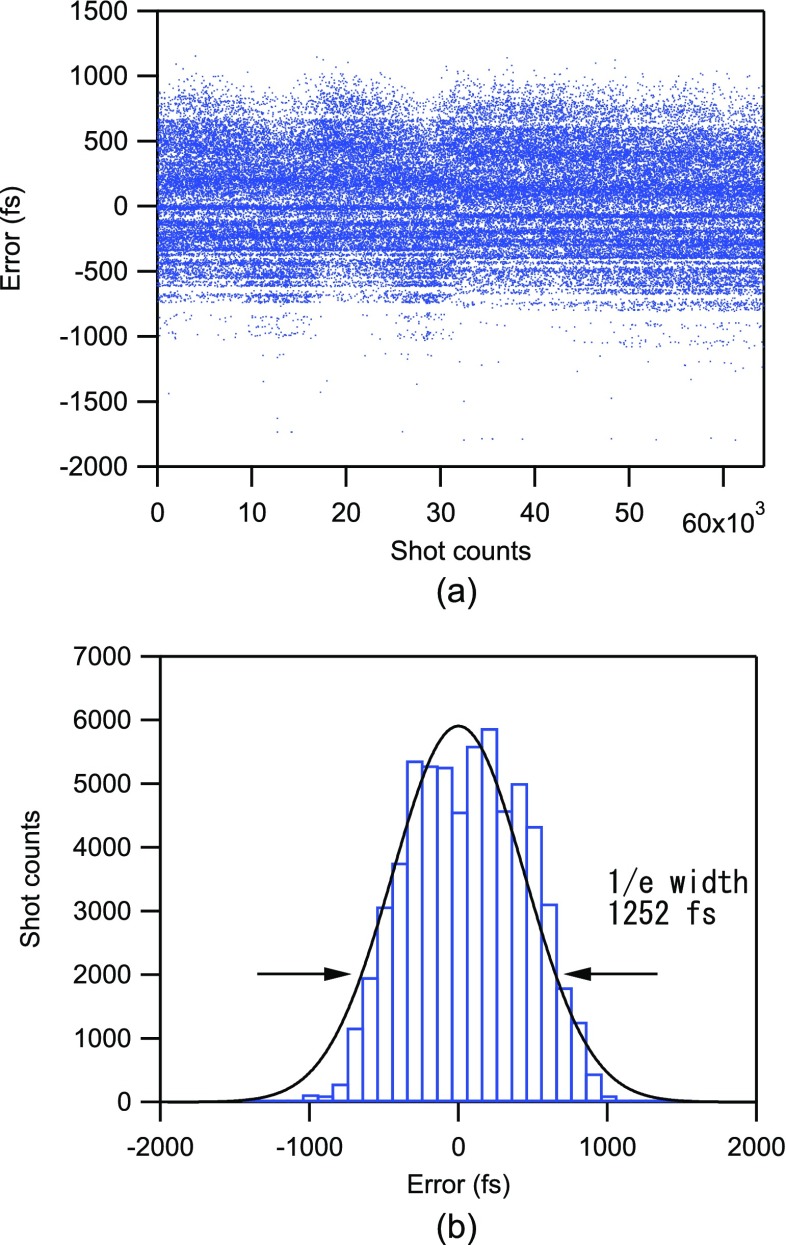
(a) Typical timing error (δτ) dataset between the UV and XFEL pulses measured by the timing monitor. (b) Histogram of the timing errors with Gaussian fitting. The 1/e width of the fitted curve was 1252 fs.

Timing diagnosis in the hard X-ray region uses a transient change in optical transmittance of a GaAs wafer under intense X-ray irradiation. Illumination of the GaAs wafer with intense X-rays creates a large number of electron-hole pairs. This alters the complex refractive index of the wafer and consequently the (detectable) transmittance for an optical laser pulse with a photon energy greater than the bandgap illuminating the wafer from an offset direction. A small portion of the fundamental laser output in the NIR was diverted before the frequency conversion for this purpose. Both the NIR and X-ray beams were focused in one dimension and were spatially and temporally overlapped on the wafer in such a way that incidence angles of optical laser pulses and X-ray pulses to the wafer are 0° and 45°, respectively. The relative timing error (δτ), originating from timing jitter, was then retrieved on a shot-by-shot basis by analyzing the spatially modulated profile of the transmitted line-shaped NIR beam. A previous two-color X-ray pulse measurement has demonstrated that the accuracy of the timing monitor was less than 16.7 fs rms.^33^ Further details of the arrival-time diagnostics system of SACLA have been described elsewhere.^27,28^ Figure 3(a) shows a typical δτ dataset used for a time-profile measurement (60 000 shots). Figure 3(b) shows the corresponding timing error histogram. The timing jitter between the XFEL and NIR pulses was estimated to be 1252 fs (full width 1/e maximum).

**FIG. 4.**
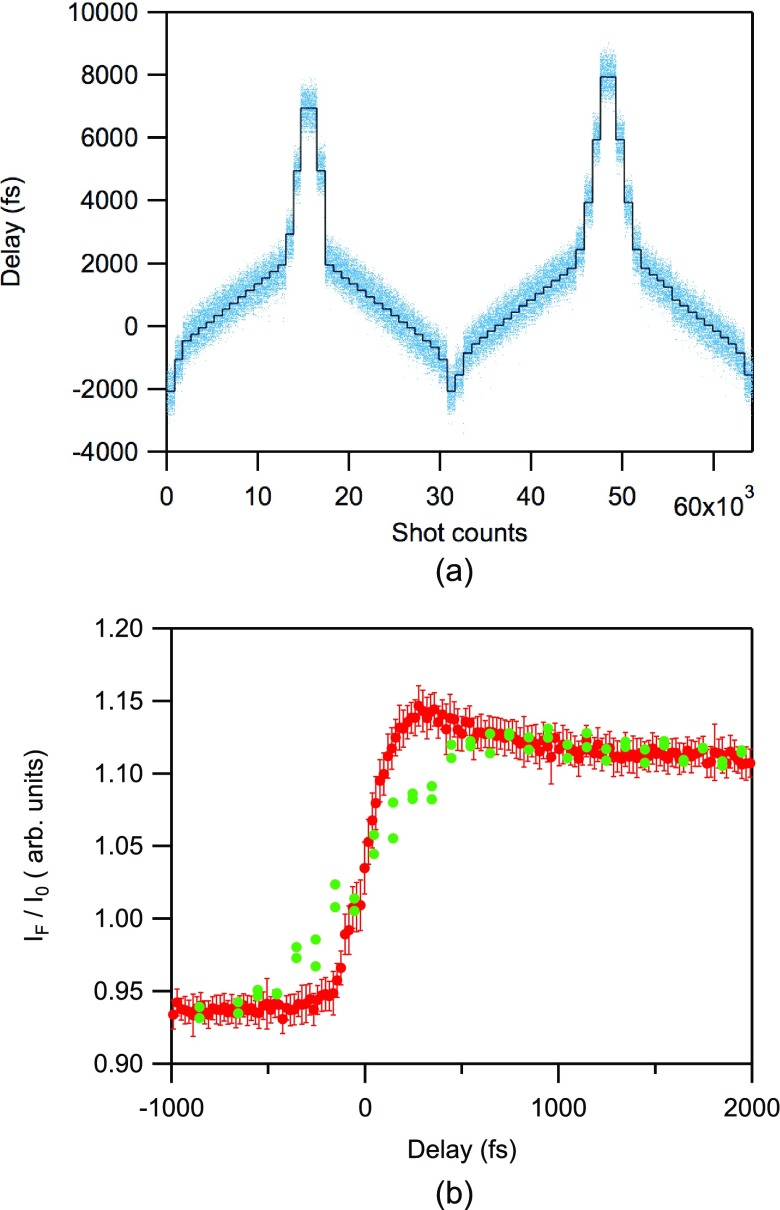
(a) The course of the fixed relative delay (τ) provided by the stage scanning (black line) and dataset of the total delay time (τ + δτ) estimated from the timing monitor data (blue dots). (b) Time profiles of the absorbance change at 4.9815 keV after timing error compensation averaged over a 20 fs window (red circles). The error bars represent the standard error of the average. For comparison, the time profile before timing correction is also shown as green dots measured with 100 fs spacing.

Using the δτ data obtained from the timing monitor, the arrival timing error in each transient profile was compensated for by the following post-processing procedure:

(1) The original dataset was collected at each delay position τ set by the translational delay stage [Fig. 4(a), black line]. The preset delay time τ and the timing error δτ were tagged for each shot, and the subsets measured at different τ were merged to generate a unified dataset using the total delay time τ + δτ [Fig. 4(a), blue dots].
(2) The unified dataset was sorted for the τ + δτ value.
(3) The absorbance was calculated from the fluorescence intensity divided by the incident XFEL intensity for each shot.
(4) The time profile was plotted [red dots in Fig. 4(b)].

The red dots in Fig. 4(b) represent the absorbance of the sorted dataset averaged with a time window of 20 fs. The green dots represent the absorbance averaged over 1000 shots at each τ with 100-fs spacing before timing correction. The difference between the red and green dots clearly shows an improvement of temporal resolution of the TR-XAS by the arrival timing monitor. The error bars on red dots represent the standard error of the average.

### E. Excitation efficiency

The parameters of the present experiment are summarized in Table I. The excitation efficiency of the sample can be expressed as

$$f=\frac{N_{\text{ph}}}{N_{A}\cdot c\cdot V}\left(1-10^{-\mu L}\right)\frac{f_{a}}{f_{a}+f_{s}}\;.$$

**TABLE I.** Experimental parameters used in the present study.

Experimental parameter		Value
Excitation laser wavelength		268 nm
Pulse energy		95 *μ*J
Focal size of UV pulse (FWHM)	Vertical	263 *μ*m
	Horizontal	262 *μ*m
Probed volume		3.1 × 10^−8^ cm^3^
Liquid jet diameter		100 *μ*m
TiO_2_ concentration		50 mM
Optical path length		100 *μ*m
Irradiated photon number in the probed volume		5.1 × 10^11^ photons
Absorption coefficient		120 cm^−1^
Scattering coefficient		19 cm^−1^
Absorbed photon number in the probed volume		3.5 × 10^11^ photons
Number of Ti atoms in the probed volume		9.5 × 10^11^ atoms
Excitation efficiency		37%

Here, *N*_ph_, *N*_A_, *c*, *V*, *μ*, and *L* are, respectively, the number of excitation light photons, Avogadro's constant, the sample concentration (*c* = 50 mM), the sample volume irradiated by the excitation light (*V* = 3.1 × 10^−8 ^cm^3^), the extinction coefficient of the sample, and the optical path length (*L* = 100 *μ*m). The extinction coefficient *μ* is the sum of the absorption coefficient *μ_a_* and the scattering coefficient *μ_s_*. *f_a_* and *f_s_* are the fractions of the loss factors, which are related to the coefficients as log_10_(1 – *f_a_*) = −*μ_a_L* and log_10_(1 – *f_s_*) = −*μ_s_L*, respectively.

The absorption and scattering of our sample can be approximately described by the Rayleigh scattering theory given the size of agglomerated nanoparticles [the mode diameter of 30 nm, measured by DLS (Fig. 1)], the wavelength (268 nm), and the refractive index of the surrounding water (1.37) (see supplementary material for details). Assuming Rayleigh scattering with a complex refractive index of 3.0–1.6i (for a randomly oriented anatase TiO_2_^34^), the absorption and scattering cross-sections are estimated to be *σ_abs_* = 3.8 × 10^−16^ m^2^ and *σ_scat_* = 5.9 × 10^−17^ m^2^, respectively. From these cross-sections and the number of agglomerated particles per unit volume (7.3 × 10^19^ m^−3^), the corresponding absorption and scattering coefficients are calculated to be *μ_a_ *= 120 cm^−1^ and *μ_s_ *= 19 cm^−1^, respectively. We note that the estimated scattering strength of the beam path (19 cm^−1^ × 0.01 cm = 0.19) is still in the single-scattering regime. The energy loss measured in the transmitted beam is therefore dominated by absorption and single scattering, whereas multiple scattering, which typically causes measurement errors in colloidal solution, is negligible in this case.

Using these parameters and assuming a Gaussian beam intensity profile of the UV pulse, we calculated the excitation efficiency of the sample. We obtained the excitation efficiency of *f* = 37% for the used excitation pulse energy of 95 *μ*J.

## III. RESULTS

### A. Steady-state absorption spectra

**FIG. 5.**
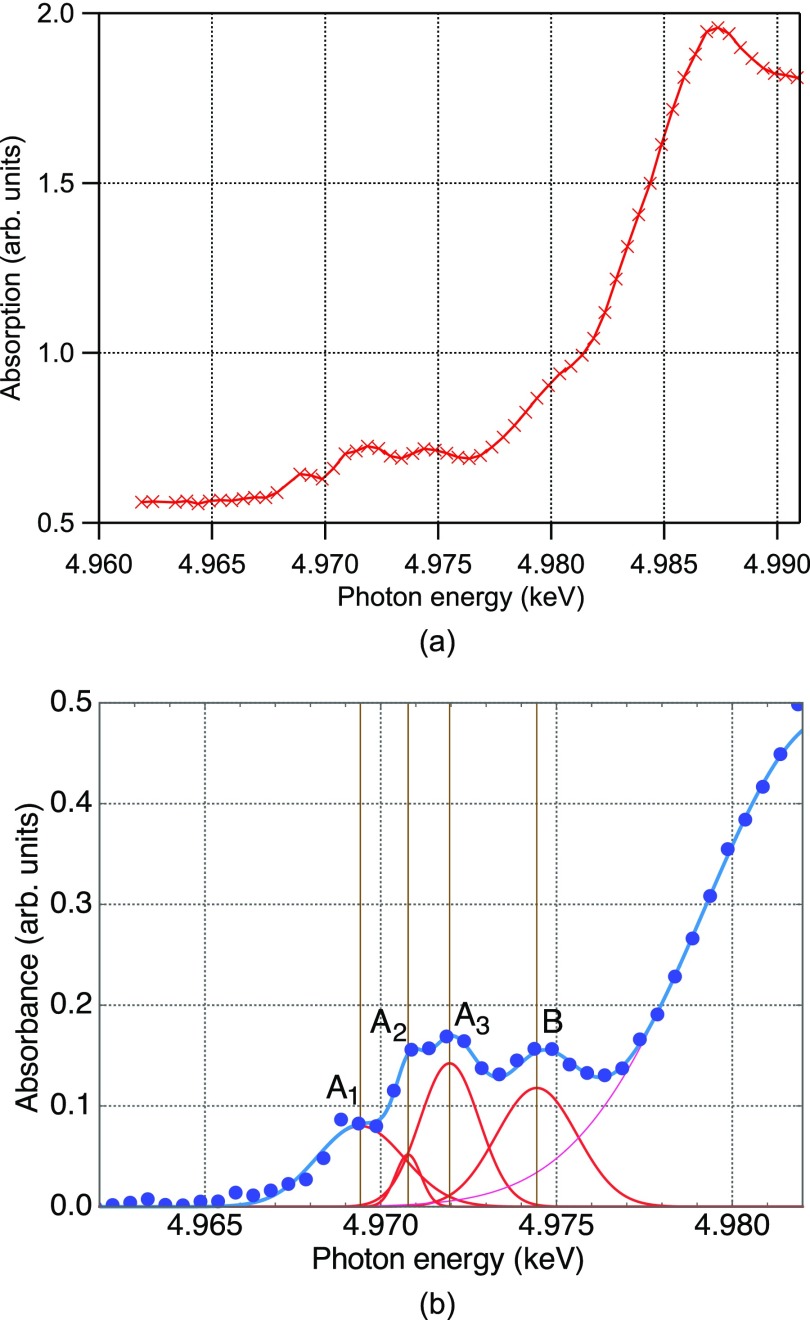
(a) Steady-state Ti K-edge X-ray absorption spectra of the sample. (b) Magnified spectrum in the pre-edge region. Blue dots are experiment data, and lines are Gaussian fitting results for the A_1_, A_2_, A_3_, and B peaks.

Figure 5(a) shows the static X-ray absorption spectrum of our sample measured at SACLA in the vicinity of the Ti K-edge (4.982 keV). This spectrum is in excellent agreement with that of TiO_2_ anatase nanoparticles previously measured using synchrotron radiation^9^ (a comparison of these two spectra can be found in the supplementary material). Figure 5(b) shows the least squares fitting of the pre-edge region of our spectrum, which reveals four peaks labeled A_1_, A_2_, A_3_, and B. The photon-energy axis here was calibrated by adjusting the A_3_ position to agree with the literature values, with which all the peak positions were in agreement with previous studies as summarized in Table II.

**TABLE II.** Positions of the pre-edge peaks, as determined by the curve fit, in comparison with the previously reported values.

	Rittmann-Frank^9^	Luca^35^	Zhang^36^	This work (keV)
A_1_	4.969	4.9688	4.9687	4.9694
A_2_	4.971	4.9709	4.9708	4.9708
A_3_	4.972	4.9719	4.9719	4.9720
B	4.974	4.9743	4.9742	4.9744

### B. Excitation intensity dependence

**FIG. 6.**
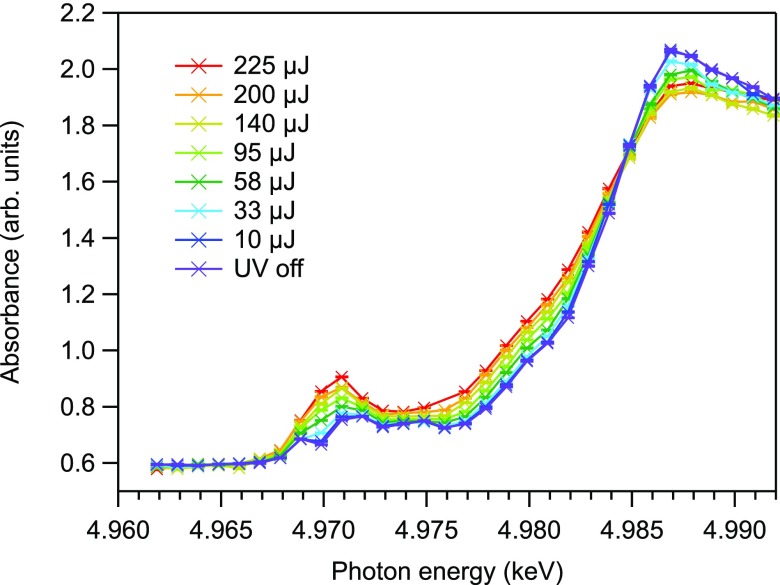
Observed transient Ti K-edge X-ray absorption spectra at 100 ps after pump irradiation for several excitation intensities. The peak absorbance at around 4.987 keV decreased with the increasing excitation intensity, while an overall increase of pre-edge features was observed.

**FIG. 7.**
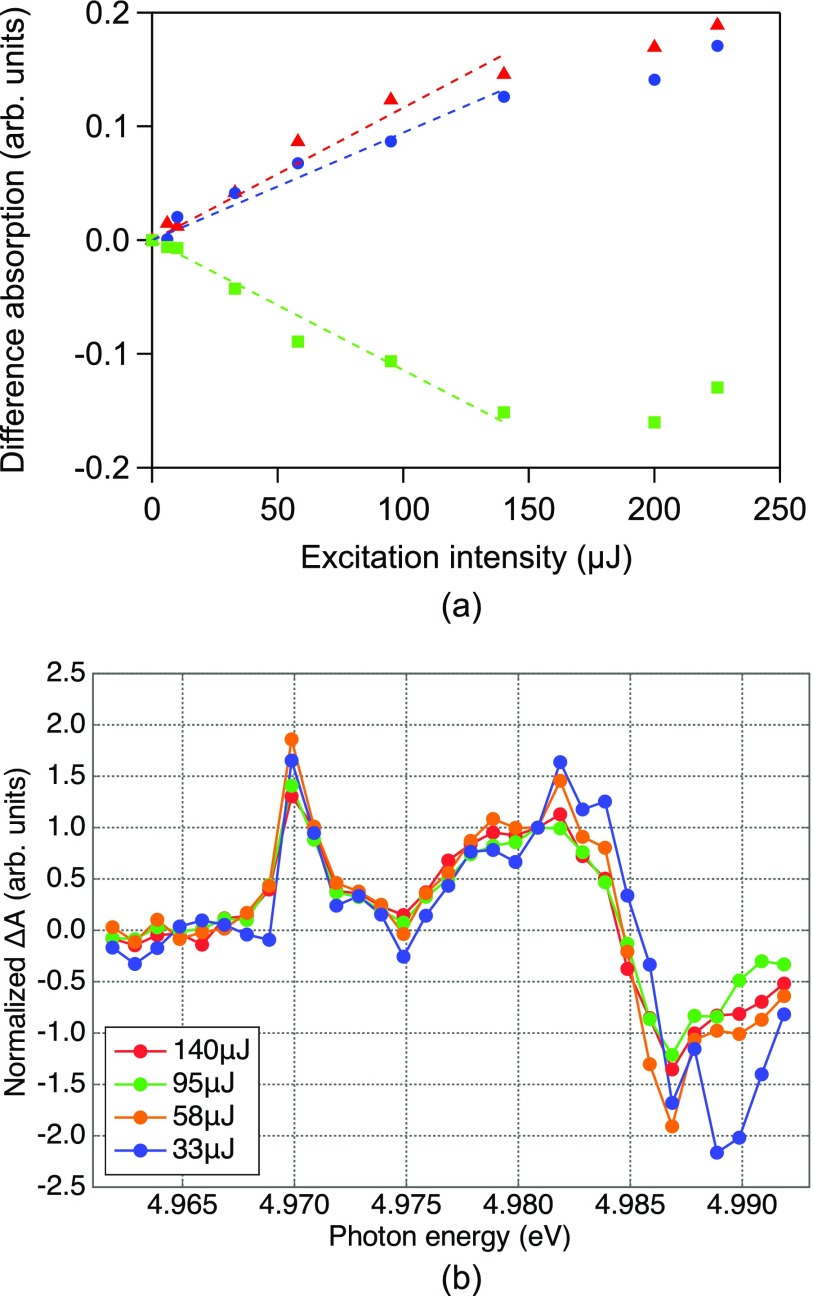
(a) Change of absorbance at 4.9695 keV (pre-edge), 4.9815 keV (edge), and 4.9865 keV (above-edge) as a function of the UV excitation intensity. The absorbance shows a linear dependence up to 140 *μ*J. The dashed lines are a linear fit of each dataset within 140 *μ*J. (b) Normalized difference absorption spectra excited at 140, 95, 58, and 33 *μ*J/pulse. The spectra exhibit identical features up to 140 *μ*J, which indicates that multiphoton effects are unimportant up to at least this intensity.

Figure 6 shows the X-ray absorption spectra measured at 100 ps for different UV light intensities. UV excitation induced a red-shift of the K-edge, which indicates that a photoreduction of Ti^4+^ to Ti^3+^ has occurred. A broadening of the edge is also observed, explained by changes of bond lengths around Ti sites in the photoexcited state.^9^ A similarly broadened profile can be observed in amorphous TiO_2_.^35^ In previous picosecond XAS measurements on TiO_2_, the observed transient spectra were explained using a red-shifted spectrum of amorphous TiO_2_, with which the reduction shift was evaluated as 0.5 to 1.0 eV.^37,38^ We also observe a spectral change in the pre-edge region, particularly around the A_2_ position. All these spectral changes are enhanced with increasing UV intensity. The quantitative UV pulse intensity dependence of the absorbance changes at 4.9695 keV (pre-edge), 4.9815 keV (edge), and 4.9865 keV (above-edge) is plotted in Fig. 7(a), which reveals a linear dependence up to 140 *μ*J. Figure 7(b) shows difference spectra at representative UV pulse intensities obtained by subtracting the static X-ray absorption spectrum of the sample solution (UV off in Fig. 6) from the transient absorption spectra (also from Fig. 6). The intensities have been normalized at 4.981 keV. The spectra exhibit identical features up to 140 *μ*J, indicating that multiphoton effects are unimportant up to at least 140 *μ*J.

### C. Time-resolved X-ray absorption spectra

**FIG. 8.**
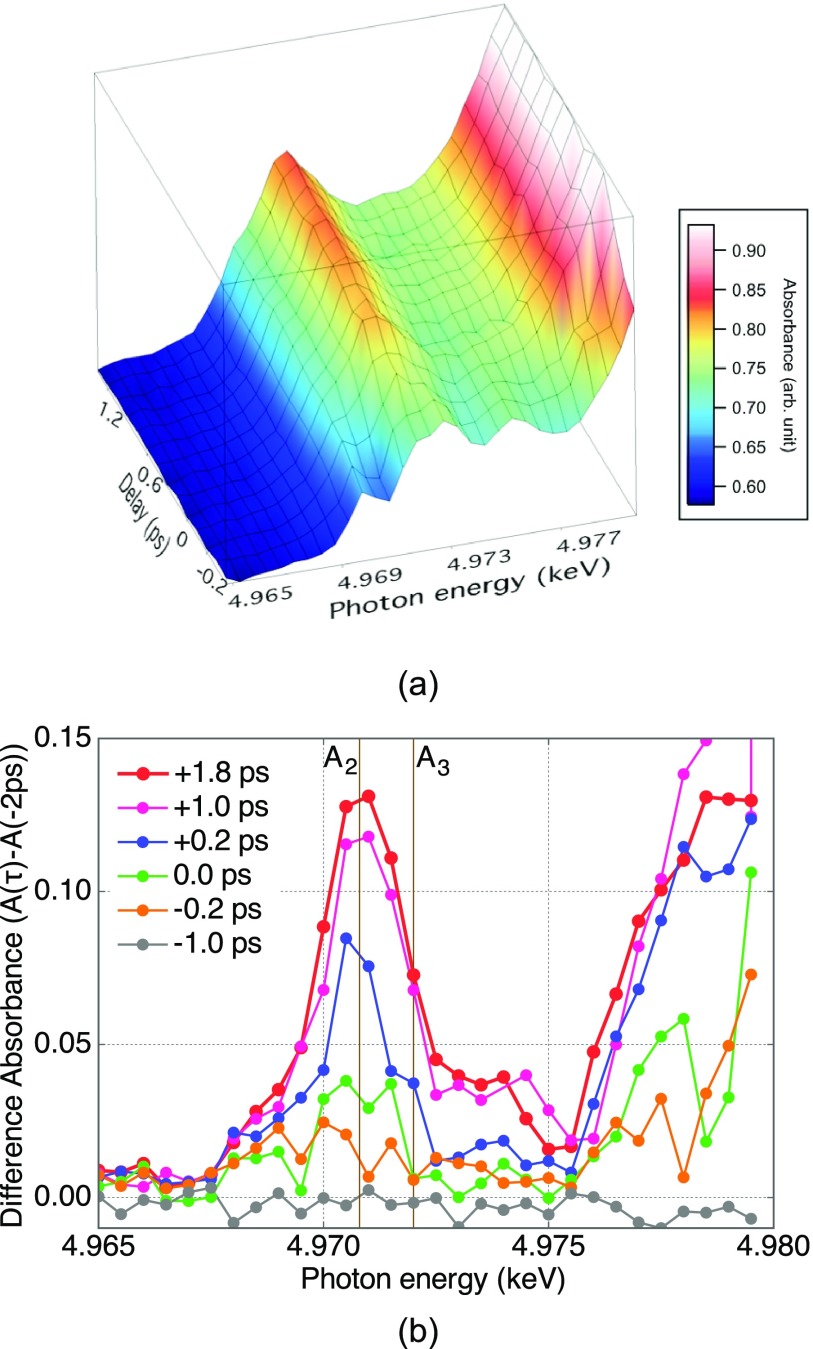
(a) Time-resolved X-ray absorption spectra and (b) their corresponding difference spectra of TiO_2_ in the pre-edge region. To extract the difference absorbance spectra, we subtracted the spectrum at –2.0 ps delay. It gets apparent that the absorbance at around A_2_ peak gradually increases after UV irradiation.

Figure 8 shows the evolution of the transient signal as a three-dimensional color-plot of the time-resolved X-ray absorption spectra in (a) and as difference spectra at selected time delays in (b). The difference spectra are obtained by subtracting the spectrum measured at –2 ps from each time-resolved excited spectrum. In Fig. 8(b), we see two distinct features: A K-edge shift to lower photon energy almost instantaneously around zero delay and a prominent peak grows near the position of A_2_ with a finite rise time.

Using the time-resolved pre-edge spectra at the delay times of –2 and +2 ps, the spectrum of the excited state can be extracted by assuming an excitation efficiency *f*. The transient absorption spectrum *A*_transient_ observed in the experiment is expressed as follows:

$$A_{\text{transient}}\;=\;fA_{\text{ex}}+\left(1-\;f\right)A_{\text{nonex}},$$

where *A*_ex_ and *A*_nonex_ are the absorption spectra of the excited and non-excited states, respectively. By substituting $A_{\text{nonex}}=A\left(-2\text{ps}\right)\;$ and $A_{\text{transient}}=A\left(+2\text{ps}\right)$ into the above formula, we obtain

$$A_{\text{ex}}\;=\;A\left(-2\text{ps}\right)+\frac{1}{f}\left\{A\left(+2\text{ps}\right)-A\left(-2\text{ps}\right)\right\}.$$

**FIG. 9.**
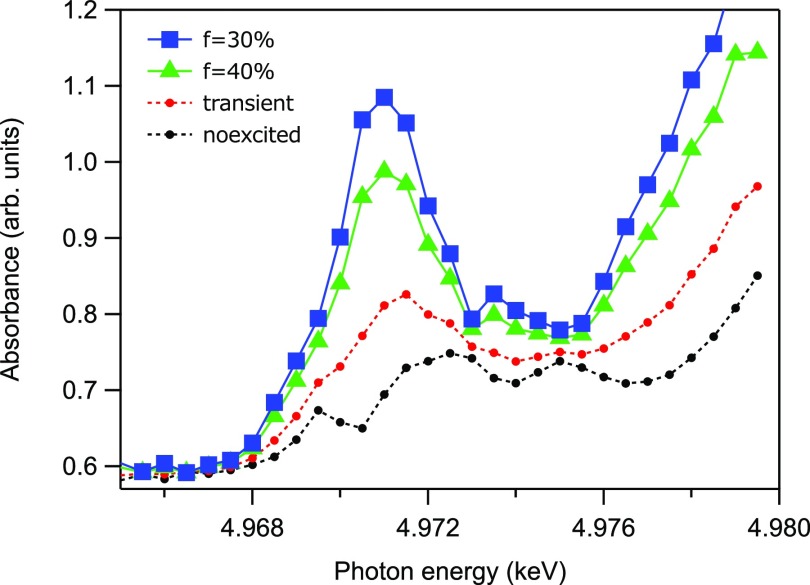
Excited-state spectra in the pre-edge region extracted by varying values of the excitation efficiency *f*. The black and red plots show the measured non-excited steady-state and excited transient spectra at 2 ps, respectively. The blue square and green triangle plots show the calculated excited-state spectra with excitation efficiencies of *f *= 30% and 40%, respectively.

The excited-state pre-edge spectra were calculated by assuming several excitation efficiencies *f* and are shown in Fig. 9. The photoexcitation efficiency has been calculated to be in the range of 30% to 40%.

### D. Temporal profiles of the absorbance change

**FIG. 10.**
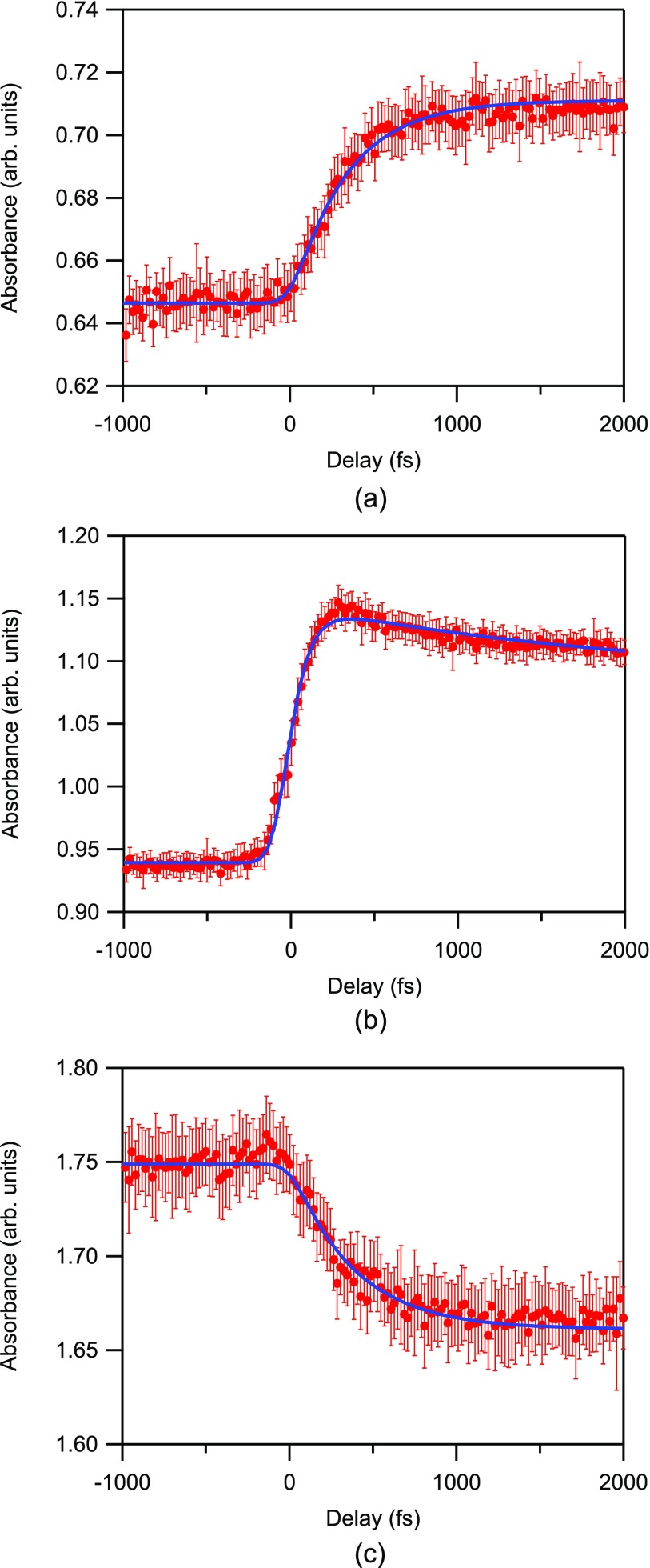
Transient profiles together with fitted decay curves at (a) 4.9695 keV, (b) 4.9815 keV, and (c) 4.9865 keV. The red dots show the absorbance of the sorted dataset averaged with a time window of 20 fs. The error bars represent the standard error of the average. The blue line is a least-squares fit to the data. Already from the curves, it is apparent that the rise time of (a) and (c) is longer than that of (b). The time constants obtained from curve fit, summarized in Table III, confirm this. The results from Table III are from a fit of the full measured range including longer delay times up to 8 ps not shown here but which are shown in the supplementary material.

In order to accurately determine the time constants for the ultrafast change of the absorption spectrum, we measured the time profiles of the X-ray absorption intensity at 4.9695 keV (low-energy side of the A_2_ peak), 4.9815 keV (edge), and 4.9865 keV (above-edge) in small time steps. Figure 10 displays the observed time profiles. The figure includes decay curves which were fitted to the full measured range from –2 to 8 ps.

The transient profiles were fitted using exponential functions while considering the instrument response function. Without the arrival time diagnostics, the timing jitter in this experiment was 1.2 ps (Full width 1/e maximum) as mentioned earlier. The instrument response function after the timing compensation was limited by the UV pulse duration to 170 fs as described in Sec. II. The profiles measured at the edge and pre-edge revealed clearly different response times. The timescale of the transient profile above the edge is similar to that at the pre-edge. The enhanced transient absorption at the pre-edge and the reduced absorption above the edge resemble the static spectrum of amorphous TiO_2_.^35^ Thus, these features point to transient electron trapping at a disordered site within the nanoparticles.

The fitting function at 4.9815 keV (edge) has an exponential rise followed by a single exponential decay component with a constant offset as expressed by

$$\left\{\left(1-e^{-t/\tau_{\text{rise}}}\right)\left(C_{1}e^{-t/\tau_{\text{decay}}}+C_{2}\right)\right\}*I(t),$$

where the symbol * denotes the convolution operator. The decay time constants in the picoseconds to nanoseconds range were reported to be 310 ps and 6 ns.^9^ Long ps-scale components cannot be determined accurately from our measurements and we set them as a constant offset within the measured window in the present study. The pre-edge and above-edge time profiles do not seem to have a fast decay component, and hence, they are expressed as

$$C\left(1-e^{-t/\tau_{\text{rise}}}\right)*I(t).$$

Here, I(t) denotes the instrument response function which was assumed to be a Gaussian. The zero delay of UV and XFEL pulses is determined from the curve fitting using the time zero as an adjustable parameter. The determined time constants are summarized in Table III.

**FIG. 11.**
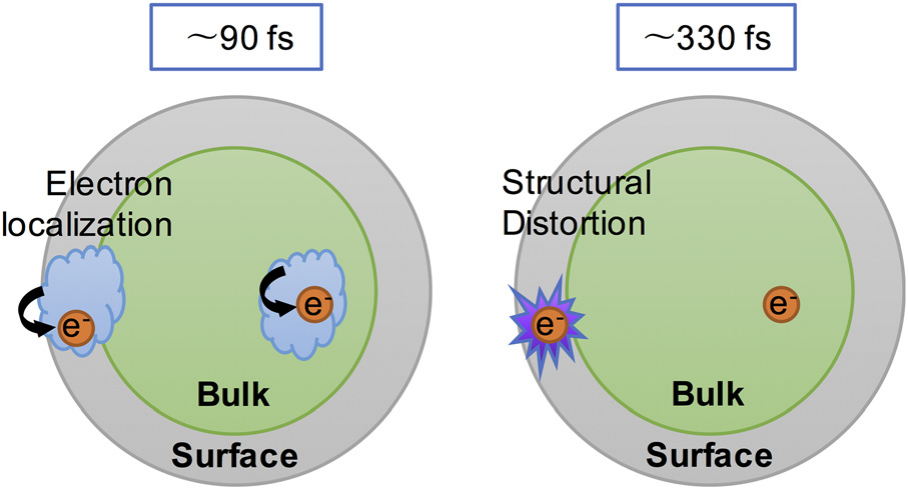
Schematic illustration of carrier and structural distortion dynamics in an anatase TiO_2_ nanoparticle. Electrons in the conduction band generated by the UV photoexcitation are localized at bulk or surface trapping sites within 90 fs. The structural distortions near the surface occur on a different timescale of 330 fs.

**TABLE III.** Time constants in picoseconds determined from the curve fitting for the data shown in Fig. 11.

	Pre-edge	Edge	Above-edge
Rise time	0.33 ± 0.02	0.09 ± 0.02	0.37 ± 0.04
Decay time	…	2.03 ± 0.02	…

Using the 170-fs temporal resolution, the rise times for the pre-edge and above-edge were determined to be 330 ± 20 fs and 370 ± 40 fs. Judging from the obtained time constant twice as large as the resolution, we are confident that this time constant is clearly resolved.

In addition to this, we further investigated the kinetics of the edge shift and have obtained a non-zero, finite time constant of 90 ± 20 fs. Even though this result is shorter than the instrumental response, we argue that this rise time is attributable to the response of the sample. To validate this hypothesis, we evaluated the χ^2^ values of the fitting data around the time zero (within ±1 ps, 166 data points). The χ^2^ values at the best fit were χ^2^ = 19 with the finite rise time and χ^2^ = 41 without the rise time, respectively (the comparison is shown in the supplementary material). We also evaluated the F ratio between the two curve-fitting models and found that the simpler model without the finite rise time (the null hypothesis) was statistically rejected (*F* = 92, *p* < 0.001). From this result, we are confident that our model based on the finite rise time is reasonable and that the time-resolved measurement has successfully resolved the short rise time of the edge shift. A previous femtosecond XAS experiment by Santomauro *et al.* suggested that such an edge-shift occurs in less than 300 fs (most likely within 170 fs).^10^ The present result indicates that the time constant is even smaller than their estimate and most likely about 100 fs.

## IV. DISCUSSIONS AND CONCLUSION

Photoexcitation of TiO_2_ promotes an electron from the valence band to the conduction band to create an exciton, which is followed by charge separation and formation of self-trapped polarons at lattice sites.^38,39^ Di Valentin and her colleagues performed density functional theory calculations on anatase TiO_2_ nanoparticles^40,41^ and identified shallow (delocalized) and deep (localized) self-trapping states of electrons; the energies of shallow traps are 0.04–0.20 eV lower than those of the conduction band edge, while energies of deep traps were <0.4 eV lower than those of the edge. The deep traps were located at either penta-coordinated Ti at the surface (faceted particles) or hexa-coordinated Ti at subsurface levels (spherical particles). The penta-coordinated Ti sites near the surface are energetically lower than those in the bulk because of a less constrained structural deformation. Therefore, it is generally favorable for the electrons to localize at the surface.

The almost instantaneous K-edge shift within about 90 fs indicates that electrons in the conduction band are localized very rapidly by reducing a Ti^4+^ lattice site to form Ti^3+^. The edge shift in this study suggests that almost one full electron charge is localized around Ti to form Ti^3+^,^9,10^ which is consistent with theoretical prediction that ∼80% of the charge is localized at a single self-trapped Ti site.^38^ Also, such ultrafast trapping was also found experimentally in an analogous system. Uemura *et al.* have performed TR-XAS of Tungsten Trioxide (WO_3_) nanoparticles using SACLA.^42^ They used 400 nm light to promote an electron from the valence band, primarily of O(2p) character, to the conduction band composed of W(5d) orbitals and monitored the transient X-ray absorption at the W L_III_ edge. They found an immediate depletion and enhancement of absorption around the edge, which were attributed to a redshift of the L_III_ edge due to electron trapping at a W^6+^ site. After the initial dynamics, structural change of the trap site was observed within 140 ps. We argue that ultrafast electron-trapping in TiO_2_ is phenomenologically similar to the case of WO_3_. Our study uncovered the delayed appearance of the pre-edge peak indicating that both the electron-trapping and structural change in TiO_2_ are rather fast and occur on a femtosecond time scale.

The pre-edge transition is attributed to structural distortion at the penta-coordinated site near the surface.^10,35^ The enhancement of the pre-edge peak is due to a 3d/4p mixing caused by structural distortion from the octahedral symmetry. Formation of hexa-coordinated polarons was shown theoretically to leave the pre-edge peak unaffected because distortion around the hexa-coordination does not break the local symmetry.^38^ We argue that the enhancement of the pre-edge peak occurs most likely near the surface, where structural deformations are less hindered at the penta-coordinated site to break the octahedral symmetry.

Electrons are localized within a certain distance from their origin of creation dictated by the diffusion length. The diffusion length within a transit time of τ can be calculated by $l\approx \sqrt{D\tau }$. The diffusion coefficients of the electron and the hole have been reported to be $D_{e}<1\times 10^{-6}$ m^2^ s^−1^ and $D_{h}<4\times 10^{-5}$ m^2^ s^−1^, respectively.^15,28^ From the edge-shift rise time of 90 fs, the diffusion length until electron localization is estimated to be l≈0.3  nm, which is similar to that reported in previous TAS^15,18^ and TR-XAS^10^ studies. One may speculate that the self-trapped polarons diffuse into a penta-coordinated site. However, the diffusion length of self-trapped polarons is theoretically predicted to be much less than 0.1 nm because the polaron is associated with slow lattice distortion.^43^ Therefore, it seems unlikely that diffusion of polarons play any important role in the femtosecond dynamics in TiO_2_.

The prominent absorbance change observed in the pre-edge region indicates that (1) a large number of penta-coordinated Ti^3+^ sites preexist in the sample due to high surface-to-bulk ratios of the small-sized nanoparticles and (2) a considerable fraction of the free electrons generated near the surface is trapped at the penta-coordinated Ti^3+^ sites. First, we estimate the ratio of the number of trap sites in the surface layer to the total number of Ti sites. We assumed the primary particle as a cube of 7 nm on each side. Bulk anatase TiO_2_ has a tetragonal crystal structure with four Ti atoms in a unit cell. The unit cell dimensions have been experimentally determined as *a *=* b *=* *0.3784 nm and *c *=* *0.9515 nm. The number of unit cells inside the cube and the ones residing at surface are estimated to be 2500 and 1100 cells, respectively. This estimation reveals that half of the unit cells are present at the surface. On the other hand, the excitation efficiency in our experiment was evaluated between 30% and 40%. On the basis of these estimations, we conclude that, even if the conduction band electrons are almost uniformly distributed within each primary nanoparticle just after photoexcitation, a large fraction of these excited electrons are in the vicinity of the surface. Furthermore, the near field in nanoparticles (the primary particle size of ∼7 nm in our case) may not be characterized by a simple attenuation length; a recent FDTD simulation revealed considerable enhancement of the electric field on the surface of 5 nm TiO_2_ nanoparticles.^44^ Similar field enhancement near the surface is expected to induce stronger UV excitation than in the bulk, which would lead to trapping of a large fraction of electrons at penta-coordinated Ti sites.

Another important finding in the present paper is that time constants are very similar for the reduction of the above-edge peak intensity (300–400 fs) and enhancement of the pre-edge peak (330 fs). Both of these spectroscopic changes are indicative of structural deformation. Thus, our results strongly suggest that the pre- and above-edge signals originate from the same structural dynamics near the surface of the nanoparticles.

Summarizing the interpretation described above, Fig. 11 explains our proposed model of the carrier dynamics and structural distortion in an anatase TiO_2_ nanoparticle. The electrons promoted into the conduction band by UV photoexcitation are localized at the trapping sites in the bulk or near the surface within 90 fs, followed by structural distortion at the penta-coordinated sites near the surface within 330 fs. Both the self-trapped polarons and the penta-coordinated species form Ti^3+^, which appear in the X-ray absorption spectrum with a similar K-edge shift. Since the subsequent structural distortion does not alter the edge shift, the edge-shift is decoupled from the structural dynamics causing the pre/above-edge signal evolution.

As for the hole dynamics, the diffusion length is l≈2  nm, which is on the same order as the primary particle radius of the stock solution. The photogenerated holes are expected to be trapped directly at the surface of the primary particles within secondary agglomerates.^31,32^ We assume that the hole trapping is negligible for the interpretation of the dynamics of the Ti K-edge and pre-edge regions.

Note that our nano-particles vary in shape. As a consequence, the deep trapping sites may involve both penta-coordinated Ti and hexa-coordinated Ti sites, according to theoretical simulations which predict different trapping sites for facets and spherical particles. It has been theoretically predicted that the carrier trapping dynamics of anatase TiO_2_ also depends on hydroxylation of the surface. However, we observed essentially the same results for three solutions prepared at different pH values (see supplementary material).

Because of the relatively low sensitivity of TR-XAS, we employed conditions for a high excitation efficiency of the nanoparticles. This created a large number of carriers in the bulk, which may have caused more rapid recombination of the carriers already on a picosecond time range. The influence of photoexcitation intensity on the excitation dynamics is to be discussed in a future work.

In conclusion, we studied the electron trapping dynamics in anatase TiO_2_ nanoparticles in an aqueous solution by femtosecond time-resolved X-ray absorption spectroscopy using an X-ray free electron laser (SACLA) in combination with a synchronized ultraviolet femtosecond laser. By applying arrival-time diagnostics of X-ray probe pulses, we achieved a time-resolution of 170 fs, which is limited by the temporal duration of the UV pulses. We observed an ultrafast Ti K-edge shift and the distinct rise of the pre-edge peak feature. The edge shift is ascribed to trapping of the conduction band electrons into self-trapped polarons or penta-coordinated Ti sites. The rise times for the growth of the pre-edge peak and the reduction of the above-edge peak were experimentally determined to be in the range of 300–400 fs (with the aid of the arrival-time diagnostics), which both correspond to the structural distortion dynamics at the penta-coordinated sites near the surface.

## V. SUPPLEMENTARY MATERIAL

See supplementary material for the detailed description about the sample preparation and characterization and calculation of the excitation efficiency by the Rayleigh scattering theory. The temporal profiles of the absorbance change in the full time range are also included.
